# Application of the WHO Workload Indicators of Staffing Need (WISN) method to estimate workforce requirements in Italian Local Health Unit Prevention Departments

**DOI:** 10.1186/s12913-026-14874-8

**Published:** 2026-06-05

**Authors:** G. Colarusso, R. Pellicanò, S. Smeraldo, A. Esposito, A. Giannoni, F. Gargano, O. Paciello, L. Baldi

**Affiliations:** 1 Experimental Zooprophylactic Institute of Southern Italy, Naples, Italy; 2 Local Health Unit of Caserta, Caserta, Italy

**Keywords:** Local Health Units (LHUs), Staffing requirements, Animal health, Food safety, U.I.P. (Unit of Individual Performance), U.P.S. (Unit of Structural Performance), Human resource planning

## Abstract

**Background:**

This study presents the application of the World Health Organization’s “Workload Indicators of Staffing Need” (WISN) methodology to the Prevention Departments of Local Health Units (LHUs) in Italy, with a focus on the Campania Region. These departments are responsible for essential public health activities, particularly food safety and animal health, which differ significantly from traditional healthcare settings because of their decentralized and inspection-based nature.

**Methods:**

The Campania Region developed a performance-based staffing model using WISN principles, introducing two key units, the unit of individual performance (U.I.P.) and the unit of structural performance (U.P.S.), to quantify the actual workload and staffing needs. The U.I.P. was defined as the time required for an operator to perform a simple inspection (4 h, including ancillary activities), while the U.P.S. was calculated by aggregating U.I.P.s at structure level and applying correction factors (such as the legal requirement to perform inspections in pairs), representing an original adaptation of the WISN approach to the Italian veterinary and food safety prevention setting. The model accounts for institutional duties, ancillary tasks, territorial logistics, and regulatory constraints such as the need for paired inspections.

**Results:**

Applied to 2022 data, the system identified a substantial staffing shortfall, with an estimated overall deficit of approximately 76 full-time equivalents (FTEs) across the Campania LHUs, ranging from about 18 missing FTEs in some urban LHUs to over 38 FTEs in predominantly rural or livestock-oriented areas. The most critical gaps were consistently observed in the animal health sector (SA), which concentrated the majority of missing FTEs and highlighted a structural shortage of veterinary staff dedicated to animal health activities.

**Conclusions:**

The model, acknowledged by the Italian Court of Auditors as an excellent tool for rationalising food safety and veterinary public health services, offers an evidence-based, scalable solution for optimising human resource allocation. Its success depends on continued data integration, training, and formalization through regional and national agreements. This approach lays the groundwork for more efficient, accountable, and transparent management of public health personnel.

## Introduction

In Italy, food safety and animal health fall under public health responsibilities and are ensured by the National Health Service (SSN) through specific territorial units organized within the Departments of Prevention [7].

In Italy, Prevention Departments are responsible for a wide range of public health functions, including the planning and delivery of official controls, surveillance, and risk-based inspections in the areas of veterinary public health and food safety. Within these departments, dedicated services carry out on-site inspections, audits, and enforcement activities in food establishments and farms to ensure compliance with national and European legislation.

The Regional Health System is based on identifying health objectives achievable through effective, measurable, and plannable actions [29]. This process requires significant commitment from public health operators, whose contributions are central to service delivery. Activity planning, and consequently the performance volumes of local health units (LHUs), must be conducted at the territorial level to ensure efficiency and resource optimization [10, 12, 13, 21].

Current legislation entrusts the regions with the task of establishing uniform criteria for defining minimum objectives both for the individual public health operators and for the operating units of which they are a part (individual and structural performance) [24].

This study focuses on the activities of the veterinary and food safety services operating within the Prevention Departments, specifically those responsible for animal health, food safety of products of animal origin, food safety of products of non-animal origin, and zootechnical production.

However, identifying such criteria is complex, especially in areas such as the Department of Prevention, where service offerings differ from those of other healthcare settings. Unlike hospital or district services, where users directly access facilities, veterinary services (SVs) and food hygiene and nutrition services (SIANs) primarily deliver services externally at establishments subject to inspection and control. This entails significant logistical and organizational implications (e.g., transportation needs, travel times) that must be considered when defining workloads [30].

Therefore, there is a need to define a reference model for evaluating individual and structural performance, in line with current regulations on healthcare personnel employment. This model should include a minimum level of qualitative‒quantitative performance assignable to individual operators (managers and support staff), which is consistent with institutional duties [28].

Considering that the Ministry of Health has not yet issued national guidelines for defining minimum performance standards, nor has an agreement been reached with regional trade unions, the Campania Region has initiated an experimental approach on the basis of the principles of the workload indicators of staffing need (WISN) method proposed by the World Health Organization (WHO) [30, 31].

At national level, this regional model has already attracted institutional attention. In the most recent report to the Italian Parliament on the management of regional health services, the Court of Auditors described the Campania approach as an excellent declination of the process of rationalising and improving food safety and veterinary public health services, highlighting its capacity to adequately support the identification of workforce needs and to establish a concrete correlation between the definition of essential levels of care, the quantification of each activity or programme, and the resources required for their implementation [6].

The aim of this research is to describe the regional WISN-based staffing model and to provide an empirical example of its application by comparing the structural performance units required to meet planned objectives with those available, in order to estimate the staffing gaps (FTEs) across Local Health Units and areas of competence [17].

## Materials and methods

To construct the model considering all relevant variables, a regional working group comprising senior territorial managers, senior regional managers, national collective agreement experts, and system management experts was established [14]. The group operated according to the eight steps outlined in the WISN Method [5, 33].

The methodological process was systematically aligned with the standard WISN steps, as shown in Table 1.

**Table 1:** WISN adaptation

WISN Step	Adaptation in this study
1. Priority cadres & facility types	Veterinary/medical managers, technicians
2. Available Working Time (AWT)	1,454 h managers; 1,420 h technicians (post non-productive time)
3. Workload components & standards	4 control types; U.I.P. = 4 h simple inspection (Table 2)
4. Performance standards	Annual U.I.P. capacity: 364 (managers), 355 (technicians)
5. Activity standards for structures	U.P.S. = Σ U.I.P. × correction factors (e.g., paired inspections)
6. Required workload	Annual control targets assigned by regional authority
7. Tolerance factors	Individual reductions (Table 2); cross-cutting factors (≤ 5%)
8. Staffing needs	FTE deficit = (Required UPS - Available UPS) / Annual U.I.P. capacity

In the Prevention Departments, three main professional groups are involved in the delivery of veterinary public health and food safety services: veterinary managers, medical managers, and prevention technicians. These professionals are jointly responsible for planning and performing official controls, conducting inspections and audits, and documenting compliance with regulatory requirements [9].

### Determine priority cadres and health facility types & estimate Available Working Time (AWT)

In the Departments of Prevention of Local Health Units, veterinary managers, medical managers, and prevention technicians operate. For each professional category, referring to the collective labour agreement [16], the theoretical annual workload was determined.

For medical and veterinary managers, the weekly working obligation is 38 h, corresponding to 1,976 annual hours. After nonworking hours for vacations, leave, midweek holidays, training, and absences were subtracted, an average effective annual total of 1,454 h per manager was obtained. In contrast, technicians have a weekly obligation of 36 h, totalling 1,420 net annual hours.

### Determine workload components and define activity standards

Regulation (EU) 2017/625 [23] stipulates that healthcare personnel can perform various official control activities, which are grouped into four types: simple inspections, complex inspections for risk categorization, audits, and remote inspections.

As there are no guidelines defining standard times for each control type, the working group developed estimates on the basis of variables such as the following:

- Complexity and diversity of control activities.
- Heterogeneity of sectors under surveillance.
- Inspection techniques employed.
- Transfer times (influenced by road conditions, distance, and topography).
- Institutional ancillary activities (e.g., public reception, training, data entry, meetings, bureaucratic tasks, judicial activities).
- Judicial police activities.
- Average times observed over a reference year.

Consequently, the unit/Inspection per person (U.I.P.) was introduced, defined as the time required for an operator to perform a simple inspection, including ancillary activities. This unit was quantified as 4 h [4].

In the absence of formal time–motion studies, the average duration of a simple inspection was estimated by the regional expert working group on the basis of their consolidated field experience and the systematic review of inspection reports and minutes recorded in the regional information system. These empirical observations were discussed until consensus was reached on a standard duration of 4 h per simple inspection, inclusive of ancillary activities.

Depending on the complexity of the control performed, the average time required increases, and the U.I.P. value adjusts accordingly (see Table 2).

**Table 2:** Average time required for different type of controls

Type of official control	Duration	U.I.*P*.
Simple Inspection	4 h	1 U.I.P.
Inspection for categorisation	6 h	1.48 U.I.P.
Remote Inspection	2 h	0.5 U.I.P.
Audit	14.8 h	3.7 U.I.P.

Dividing the available hours by 4 yields the theoretical annual number of U.I.P.s per operator: 364 U.I.P.s for medical/veterinary managers and 355 U.I.P.s for technicians [22].

### Define performance standards for structures (U.P.S.)

To calculate structural-level performance, the unit of structural performance (U.P.S.) was adopted, obtained by summing the U.I.P.s of personnel within the structure and applying corrective formulas. These formulas consider ANAC Resolution No. 72/2013 [3], which recommends that inspections be conducted by at least two operators selected through random rotation.

In the LHUs, personnel are organized into simple organizational structures within four main areas of competence: food safety of animal-origin products (IAOA), animal health (SA), food safety of non-animal-origin products (SIAN), and zootechnical production (IAPZ).

The U.P.S. thus quantifies the minimum number of inspections each organizational structure must guarantee annually. The sum of the U.P.S.s of the organizational structures determines the minimum annual performance expected from each Local Health Unit.

Official control activities are established annually by the Ministry of Health and the regional health authority concerning their strategic objectives. Each objective entails the execution of official controls in numerical and qualitative terms, formally assigned to each LHUs.

Each control activity consumes man-hours and, therefore, U.P.S., depending on the type and purpose of the control; thus, we refer to necessary U.P.S.s, the sum of man-hours required to achieve the annually assigned numerical objectives, and available U.P.S.s, the sum of man-hours each company currently has available [1, 25, 30].

Workload management—i.e., organizational charts, task identification, U.I.P., and U.P.S.—is assigned, managed, and monitored through a digital platform that oversees all official control activities named “GISA-Campania”. The entered data are visible in the information system and can be exported as needed for operational or managerial purposes.

### Determining tolerance factors and staffing needs

To determine the actual individual workload, the group defined reduction factors affecting individuals under specific conditions (additional assignments, maternity, breastfeeding, health issues, etc.). These reductions are regulated through a technical document (see Table 3) identifying topics and applicable deduction ranges.

These tolerance factors were defined by a regional technical working group on the basis of existing contractual provisions and organizational needs, with the aim of accounting for additional responsibilities, managerial roles, and specific personal conditions that reduce the effective time available for routine inspection activities [6, 8, 11].

**Table 3:** Percentage of individual reduction for different motivations

Nr	Factors	MAXIMUM % REDUCTION
1	Health reasons	Max 90%
2	Political position	10%-39%
3	Secondment to other health office	90%
4	Structure Management	80% − 95%
5	Office manager	25% + 1% for each person included (35%)
6	Regional Reference Centre Directors	80%
7	Collaborating with and/or replacing the Director	70%
8	Quality management system referent	80%
9	Contact person for Prevention Plan activities	3%
10	Other issue referent	5% for each issue*(max 15%)
11	iRASFF referent	Max 10%
12	auditor NURECU	5%
13	Technician coordinator	3%
14	Specific delegations	15%
15	Mycological inspectorate	6%
16	Head of training course	5%

These tolerance factors correspond to WISN category allowances (non-productive time for management, training, etc.) and activity allowances (extra time for complex tasks). Unlike clinical WISN (patient-focused), our adaptation:

- Includes regulatory allowances (paired inspections, ANAC 72/2013).
- Accounts for field logistics (travel, terrain) absent in hospital settings.
- Uses administrative platform data (GISA-Campania) for real-time validation.

Other cross-cutting factors that may impact individual or structural minimum workloads include the following:

- Shortage of technical-administrative staff.
- Insufficient instrumental resources.
- Logistical and morphological difficulties of the territory.

These elements can justify a reduction of up to 5% of the total individual or structural workload, as they entail additional time expenditure in performing routine activities [26].

The difference between the available and required UPSs was used to define the number of missing staff units broken down by organisational area and specific competencies [2, 18].

### Staffing gap calculation

The core mathematical logic follows:

1. Individual Capacity: Annual U.I.P.s = AWT / 4 h.
  Managers: 1,454 h / 4 h = 364 U.I.P./year.
  Technicians: 1,420 h / 4 h = 355 U.I.P./year.
2. Adjusted Individual Capacity: Annual U.I.P.s × (1 - tolerance factor).
3. Structural Capacity (Available U.P.S.): Σ(Adjusted individual U.I.P.s per structure) × 0.5 (paired inspections).
4. Required U.P.S.: Σ (U.I.P.s for assigned controls by type, Table 2).
5. FTE Deficit: (Required U.P.S. - Available U.P.S.) / Annual U.I.P. capacity.
  Example (Avellino SA): (12,017 − 1,129) / 364 = ~ 38 FTE.

## Results

A quantitative exploratory-descriptive study with a methodological intervention was conducted, following the phases outlined in the WISN method, to calculate and analyse the workload of personnel employed in prevention departments in veterinary public health and food safety activities.

Although the calculation system has been available since 2018, data have been used since 2022, as a training period for personnel was deemed necessary to ensure proper understanding and stability of the collected information.

The availability of personnel in 2022 for each LHU in the Campania Region is reported in Table 4.

**Table 4:** Distribution of staff unit by role

LHUs	Tot	Veterinarians	Physicians	Technicians
Avellino	66	48	9	9
Benevento	105	81	10	14
Caserta	350	279	20	51
Napoli 1 Centre	143	76	33	34
Napoli 2 North	129	82	14	33
Napoli 3 South	181	111	31	39
Salerno	283	215	23	45

LHUs: Local Health Units

The structure managers identified the personnel involved and, for each unit, defined the individual workload (U.I.P.), applying any deductions provided by the calculation system (see Table 5) [32].

The obtained U.I.P. values were aggregated for each operational area, defining the absolute available potential (U.P.S.). This value was subsequently halved in accordance with Italian anticorruption regulations, which stipulate that official control activities be conducted in pairs.

**Fig. 1:**
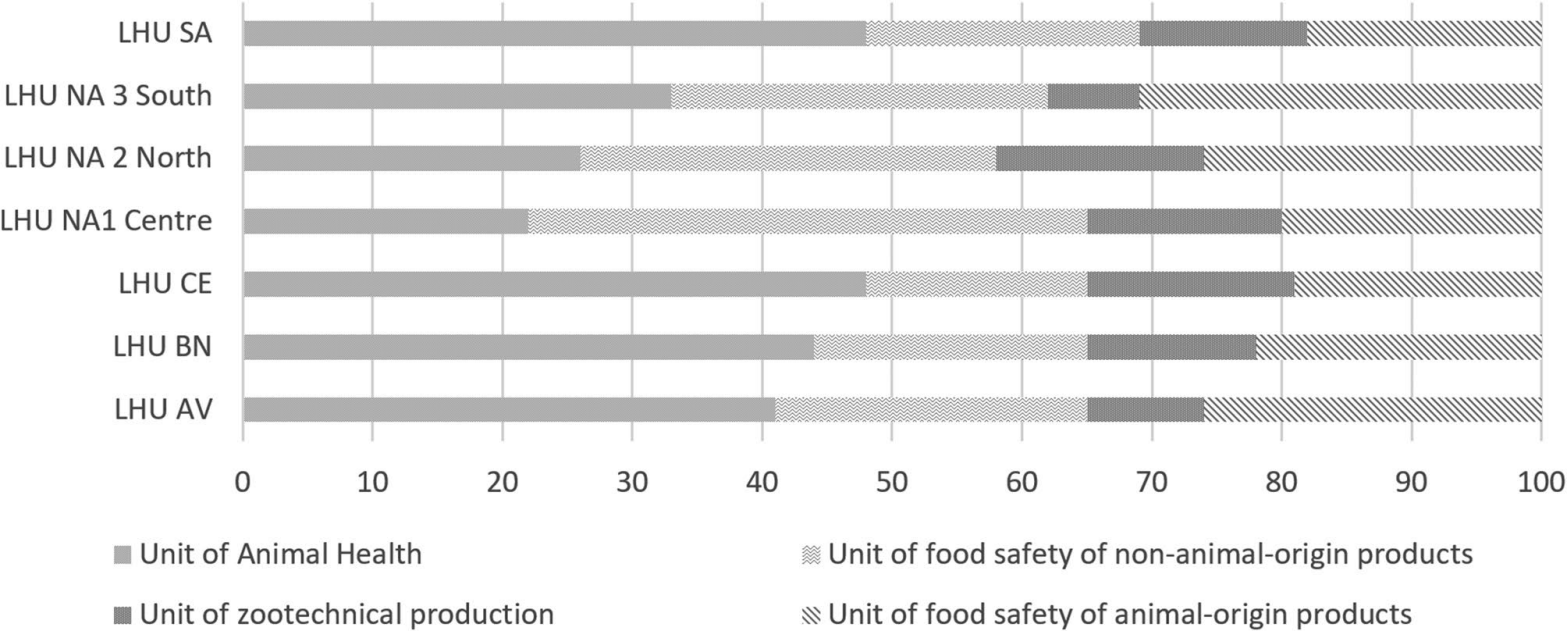
Percentage distribution of staff units by area and LHUs (Local Health Units)

Figure 1 shows the distribution of personnel by operational area. In LHUs with a zootechnical focus, over 40% of personnel are employed in animal health, whereas in LHUs with urban coverage, the majority are allocated to food safety.

**Table 5:**
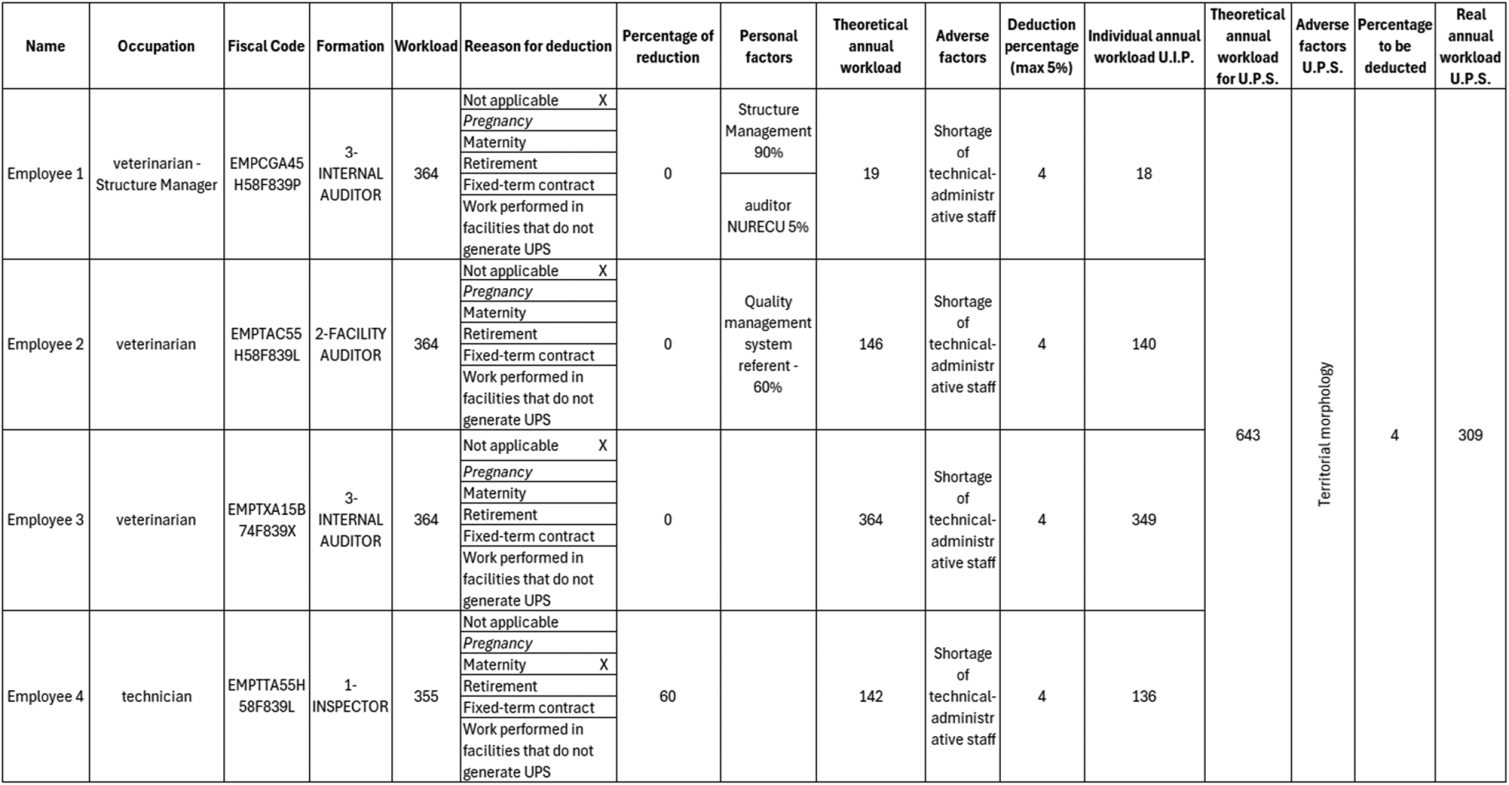
Example of output from an individual and organizational workload calculation model

Columns A, B, and C report the worker’s personal details and their role

Column D indicates the level of training

Column E shows the annual workload expressed in U.I.P. (Individual Workload Units)

Columns F and G include the upstream workload reduction factors and their respective percentages

Column H presents the reduction factors related to individual-specific conditions

Column I and L reports the calculation of the U.I.P. net of deductions

Columns J and K contain the reduction percentages due to cross-cutting factors affecting the personnel

Columns M to P display the calculation of the U.P.S. (Organizational Workload Units) of the structure

The outcomes of the calculation model are presented in Tables 6, 7, 8, 9, 10, 11 and 12, which illustrate the following:

- The available UPS (sum of the UIPs performed by personnel),
- The required UPS (needed to meet the annual targets assigned by the regional authority),
- The staffing shortfall, which is calculated as the difference between the two.

**Table 6:** LHU Avellino outcomes of the calculation model

Local Health Unit of Avellino	UPS available	UPS needed	Difference	Conversion of UPS deficit into UIP equivalent	Deficient people units
IAOA	2527	7093	-4566	-9314	-25
IAPZ	370	1642	-1272	-2595	-7
SA	1129	12,017	-10,888	-13,937	-38
SIAN	662	1670	-1008	-2056	-6
tot.	4688	22,422	**-17,734**	**-27,902**	**-76**

SA: animal health; IAOA: food safety of animal-origin products; SIAN: food safety of non-animal-origin products; IAPZ: zootechnical production; U.I.P.: Unit of Individual Performance; U.P.S.: Unit of Structural Performance

**Table 7:** LHU Benevento outcomes of the calculation model

Local Health Unit of Benevento	UPS available	UPS needed	Difference	Conversion of UPS deficit into UIP equivalent	Deficient people units
IAOA	2327	3724	-1397	-2849	-8
IAPZ	1308	1278	30	n.a.	n.a.
SA	3746	12,745	-8999	-9359	-25
SIAN	1234	1125	109	n.a.	n.a.
tot.	8615	18,872	**-10,257**	**-12,208**	**-33**

SA: animal health; IAOA: food safety of animal-origin products; SIAN: food safety of non-animal-origin products; IAPZ: zootechnical production; U.I.P.: Unit of Individual Performance; U.P.S.: Unit of Structural Performance

**Table 8:** LHU Caserta outcomes of the calculation model

Local Health Unit of Caserta	UPS available	UPS needed	Difference	Conversion of UPS deficit into UIP equivalent	Deficient people units
IAOA	9597	9385	212	n.a.	n.a.
IAPZ	6649	5455	1194	n.a.	n.a.
SA	5095	13,379	-8284	-15,001	-41
SIAN	1761	1770	-9	n.a.	n.a.
tot.	23,102	29,989	**-6887**	**-15,001**	**-41**

SA: animal health; IAOA: food safety of animal-origin products; SIAN: food safety of non-animal-origin products; IAPZ: zootechnical production; U.I.P.: Unit of Individual Performance; U.P.S.: Unit of Structural Performance

**Table 9:** LHU Napoli 1 centre outcomes of the calculation model

Local Health Unit of Napoli 1 Centre	UPS available	UPS needed	Difference	Conversion of UPS deficit into UIP equivalent	Deficient people units
IAOA	2907	3969	-1062	-2166	-6
IAPZ	1842	1511	331	n.a.	n.a.
SA	4050	7251	-3201	-3328	-9
SIAN	1577	2043	-466	-952	-3
tot.	10,376	14,774	**-4398**	**-6446**	**-18**

SA: animal health; IAOA: food safety of animal-origin products; SIAN: food safety of non-animal-origin products; IAPZ: zootechnical production; U.I.P.: Unit of Individual Performance; U.P.S.: Unit of Structural Performance

**Table 10:** LHU Napoli 2 North outcomes of the calculation model

Local Health Unit of Napoli 2 North	UPS available	UPS needed	Difference	Conversion of UPS deficit into UIP equivalent	Deficient people units
IAOA	4349	3796	553	n.a.	n.a.
IAPZ	2125	1798	327	n.a.	n.a.
SA	3441	7111	-3670	-4550	-12
SIAN	1897	2220	-323	-835	-2
tot.	11,812	14,925	**-3113**	**-5385**	**-14**

SA: animal health; IAOA: food safety of animal-origin products; SIAN: food safety of non-animal-origin products; IAPZ: zootechnical production; U.I.P.: Unit of Individual Performance; U.P.S.: Unit of Structural Performance

**Table 11:** LHU Napoli 3 South outcomes of the calculation model

Local Health Unit of Napoli 3 South	UPS available	UPS needed	Difference	Conversion of UPS deficit into UIP equivalent	Deficient people units
IAOA	6850	6246	604	n.a.	n.a.
IAPZ	1362	1494	-132	-269	-1
SA	7184	11,739	-4555	-6332	-17
SIAN	2719	2273	446	n.a.	n.a.
tot.	18,115	21,752	**-3637**	**-6601**	**-18**

SA: animal health; IAOA: food safety of animal-origin products; SIAN: food safety of non-animal-origin products; IAPZ: zootechnical production; U.I.P.: Unit of Individual Performance; U.P.S.: Unit of Structural Performance

**Table 12:** LHU Salerno outcomes of the calculation model

Local Health Unit of Salerno	UPS available	UPS needed	Difference	Conversion of UPS deficit into UIP equivalent	Deficient people units
IAOA	6156	7866	-1710	-3488	-10
IAPZ	6593	8579	-1986	-4051	-11
SA	19,599	30,056	-10,457	-10,791	-30
SIAN	2963	3521	-558	-1159	-4
tot.	35,311	50,022	**-14,711**	**-19,489**	**-55**

SA: animal health; IAOA: food safety of animal-origin products; SIAN: food safety of non-animal-origin products; IAPZ: zootechnical production; U.I.P.: Unit of Individual Performance; U.P.S.: Unit of Structural Performance

The minimum UPS requirements were determined on the basis of the number of official controls assigned by the regional competent authority to each local health unit (LHU). Each LHU, through its structural managers, allocates the number of controls to be performed across operational areas according to jurisdiction and territorial extent.

The gap between the available and required UPSs was used to estimate the number of missing personnel units.

In 2022, the Local Health Unit of Avellino experienced a significant shortfall across all operational areas, with an unmet need of 17,734 UPSs, corresponding to 76 full-time equivalents (FTEs). The most critical shortage was recorded in the Operational Unit of Animal Health (UOC SA), with deficits of -10,888 UPSs and − 38 FTEs.

The Local Health Unit of Benevento reported a considerable deficit concentrated in the UOC SA, with a shortfall of 8,999 UPSs, equivalent to 25 missing personnel units. Two operational units (IAPZ and SIAN) recorded slight surpluses.

The Local Health Unit of Caserta experienced a significant and exclusive shortage in the UOC SA, with a staffing gap of 41 FTEs.

The Local Health Unit of Napoli 1 Centre presented a relatively balanced situation, with relevant shortages only in the UOC SA (which, in an urban context, corresponds to veterinary urban hygiene activities), showing a negative balance of -3,328 UIP, equivalent to 9 FTEs. Both the Local Health Unit of Napoli 2 North and the Local Health Unit of Napoli 3 South reported staff shortages in the area of animal health, amounting to 12 and 17 FTEs, respectively.

The Local Health Unit of Salerno displayed the second most critical scenario after Avellino, with deficits of -14,711 UPSs and − 19,489 UPPs. The UOC SA recorded the highest individual shortfall, with − 10,457 UPSs, equivalent to 30 missing personnel units.

## Discussion

The staff requirement calculation system developed by the Campania Region, inspired by the World Health Organization’s WISN methodology, represents a significant step toward more rational, transparent, and measurable human resource planning within the Departments of Prevention, with reference to the Veterinary Services and SIAN.

To our knowledge, this is the first WISN-based model specifically designed and applied to veterinary public health inspection activities. A systematic literature review found no published WISN applications in this field, either in Italy or internationally, representing a substantive methodological innovation for decentralized, regulatory-driven public health services.

The adoption of the **individual performance unit (U.I.P.)** as the measure for individual activities and the **structural performance unit (U.P.S.)** for structural output has enabled the objectification of performed tasks and the direct linkage between service volumes and the actual operational capacities of the structures and professionals involved. This approach has allowed, for the first time, a systemic and evidence-based quantification of the personnel needed to achieve regional strategic objectives and national essential levels of care.

The model adopted was also assessed by the Italian Court of Auditors in its “Report to Parliament on the Management of Regional Health Services 2002–2023”, which described it as “excellent” [9]. However, this institutional appraisal should be interpreted with caution. The Court of Auditors’ judgement refers to the coherence of the model with broader processes of rationalisation and to its capacity to link essential levels of care, planned activities, and required resources, rather than to a full validation of its predictive accuracy or long-term impact. The present analysis is based on data from an early phase of implementation and remains dependent on data quality, organisational stability, and the specific regional context; additional applications over time and in other settings are needed before considering this approach as fully consolidated. Nevertheless, the Court endorsed the outcome of the monitoring of the national tables, which had words of praise for the effectiveness of the Campania system, which in turn was part of the complex process activated by the Region itself to standardize the procedures for the provision of controls and for their planning on the basis of the risk inherent in the production realities subject to official control.

The analysis of 2022 results revealed that, despite the model’s introduction, significant discrepancies remain between available and required resources, particularly in the field of animal health, with substantial differences between urban LHUs and those with a strong focus on livestock farming [27].

This evidence confirms the model’s capacity to identify critical gaps accurately, offering decision-makers a reliable tool for planning and rebalancing interventions [15].

Although the U.I.P. duration of 4 h provides a pragmatic operational standard, its empirical basis remains an approximation derived from expert consensus and administrative records rather than formal time-motion studies. Even modest variations in this core parameter would propagate non-linearly through available U.P.S., staffing deficits, and FTE calculations, representing a fundamental sensitivity that warrants formal validation.

The recording of the duration of different inspection activities is currently ongoing through automatic logging in the information system. The collection of these data over a period of at least 5 years will, in the near future, allow an empirical validation of the durations originally defined by the working group based on field experience. Similarly, the extent to which correction and tolerance factors may influence staffing deficits and FTE estimates through a degree of subjectivity could be assessed in a subsequent dedicated analysis aimed at quantifying the variability introduced by these parameters.

The multiple correction and tolerance factors enhance realism by capturing managerial roles, protected personal conditions (Law 104/1992), and structural constraints, but their cumulative application introduces complexity and potential subjectivity. In the present study, these factors were applied according to the regional technical framework, but their independent contribution to staffing deficits was not separately quantified. A dedicated methodological study will therefore be needed to isolate their specific impact and to define more precisely the range of variability that they introduce into U.I.P. and U.P.S. estimates, staffing deficits, and FTE requirements. It should also be noted that the system estimates the minimum annual workload; therefore, situations of personnel surplus are unlikely. On the contrary, where additional staff are available, Local Health Units may expand the volume of official controls, which are subject to variable annual planning according to human resource availability. Finally, although the model was developed in a regional context, its calculation framework could be applied throughout Italy, where the organizational and contractual system is largely similar, and may also provide a reference for other organizations delivering inspection-based services, provided that appropriate contextual customization is made.

Unlike most WISN applications focused on clinical settings, this model demonstrates its feasibility in decentralized, inspection-based public health services.

Unlike classical WISN applications in clinical settings—which measure patient encounters in fixed facilities—this model demonstrates feasibility for decentralized, inspection-based public health services through key adaptations: workload driven by regulatory targets (not patient demand); U.I.P. including travel/ancillary time; structural corrections for mandatory paired inspections (ANAC 72/2013); real-time data from GISA-Campania platform versus manual logs.

## Conclusion

The full effectiveness of the model will be achieved only through the following:

- The formalization of regional criteria agreed upon with trade unions to enable widespread adoption of the system;
- Continuous data integration and ongoing training of involved personnel;
- Periodic updating of the parameters used, in line with evolving legal, technological, and organizational frameworks.

In summary, the adopted system has proven to be a valuable and adaptable tool for the governance of human resources in prevention services, fostering a culture of efficiency, accountability, and transparency [19].

Its broader dissemination and stabilization represent crucial conditions for strengthening community public health and for the full implementation of regional prevention policies.

To this end, the engagement of institutional stakeholders, including health authorities and policy-makers, becomes essential to ensure a shared strategic vision [20].

Moreover, the integration of the model within digital health platforms and regional health information systems could further enhance its potential, enabling real-time monitoring and more responsive planning. Attention should also be paid to the harmonization of practices across different LHUs to avoid fragmentation and ensure equity in resource allocation.

Finally, investing in change management and promoting a participatory approach among healthcare professionals can facilitate the cultural shift needed to support this transformation. By embedding the model within a broader framework of continuous improvement and evidence-based decision-making, regions can lay the groundwork for a more resilient and sustainable public health system.

## Data Availability

The datasets analysed during the current study are publicly available and can be obtained from the corresponding author upon reasonable request.

## Abbreviations

U.I.P.: Individual performance unit
U.P.S.: Structural performance unit
UOC: Operational Unit
IAOA: Food safety of animal-origin products service
SA: Animal health service
SIAN: Food safety of non-animal-origin products service
IAPZ: Zootechnical production service
U.I.P.: Unit of Individual Performance
U.P.S.: Unit of Structural Performance
FTEs: Full-time equivalents
